# Glucose-Regulated Protein 94 (GRP94): A Novel Regulator of Insulin-Like Growth Factor Production

**DOI:** 10.3390/cells9081844

**Published:** 2020-08-06

**Authors:** Yair Argon, Sophie E. Bresson, Michal T. Marzec, Adda Grimberg

**Affiliations:** 1 Department of Pathology and laboratory Medicine, Perelman School of Medicine, University of Pennsylvania, Philadelphia, PA 19104, USA; 2 Division of Cell Pathology, Children’s Hospital of Philadelphia, Philadelphia, PA 19104, USA; 3 Department of Biomedical Sciences, University of Copenhagen, Blegdamsvej 3B, 2200 Copenhagen N, Denmark; sophie.bresson@sund.ku.dk (S.E.B.); michal@sund.ku.dk (M.T.M.); 4 Department of Pediatrics, Perelman School of Medicine, University of Pennsylvania, Philadelphia, PA 19104, USA; grimberg@email.chop.edu; 5 Division of Endocrinology and Diabetes, Children’s Hospital of Philadelphia, Philadelphia, PA 19104, USA

**Keywords:** glucose regulated protein (GRP) 94, insulin-like growth factor, obligate chaperone

## Abstract

Mammals have two insulin-like growth factors (IGF) that are key mediators of somatic growth, tissue differentiation, and cellular responses to stress. Thus, the mechanisms that regulate the bioavailability of IGFs are important in both normal and aberrant development. IGF-I levels are primarily controlled via the growth hormone-IGF axis, in response to nutritional status, and also reflect metabolic diseases and cancer. One mechanism that controls IGF bioavailablity is the binding of circulating IGF to a number of binding proteins that keep IGF in a stable, but receptor non-binding state. However, even before IGF is released from the cells that produce it, it undergoes an obligatory association with a ubiquitous chaperone protein, GRP94. This binding is required for secretion of a properly folded, mature IGF. This chapter reviews the known aspects of the interaction and highlights the specificity issues yet to be determined. The IGF–GRP94 interaction provides a potential novel mechanism of idiopathic short stature, involving the obligatory chaperone and not just IGF gene expression. It also provides a novel target for cancer treatment, as GRP94 activity can be either inhibited or enhanced.

## 1. Introduction

As primary drivers of growth and proliferation at the somatic, tissue and cellular levels, the insulin-like growth factors (IGFs) must have tightly regulated activity—in terms of amount, timing, and spatial specificity and coordination. The “somatomedin hypothsies”, the original framework of understanding IGF-I production and action, has undergone considerable development with time, as additional layers of complexity and nuance have been elucidated [1]. This review summarizes traditionally recognized regulators of IGF production and action in health and disease, and adds yet another: glucose regulated protein 94 (GPR94). GRP94, a ubiquitously expressed chaperone in the endoplasmic reticulum, is required for the proper folding and secretion of both IGFs. Although insulin, the other member of the IGF hormone family, shares similarities with the IGFs structurally (including approximately 50% amino acid homology to the IGFs), in their receptors and signaling, and is itself chaperoned by GRP94, this review will focus on the IGFs. By providing a novel nexus of regulating IGF production, GRP94 and its alterations serve as a potentially new mechanism of dysregulated growth, such as idiopathic short stature and cancer, and thereby may lead to new therapeutic interventions. 

## 2. Regulators of IGF Levels Clinically

Insulin-like growth factor (IGF)-I is made throughout the body, though ~70% of circulating levels are of hepatic origin. Clinically, measurement of circulating IGF-I concentration is used most commonly as a marker of growth hormone (GH) bioactivity. Due to the GH dependence of transcription of the genes encoding IGF-I and its principal circulating partner, IGF binding protein (IGFBP)-3, normal levels of IGF-I and IGFBP-3 generally reflect normal GH activity (i.e., exclude GH deficiency) [2,3]. Further, because serum levels of these molecules do not fluctuate diurnally as does the pulsatile secretion of GH, they serve as convenient indicators of GH secretion that are measurable on a random blood sample. Indeed, circulating concentration of IGF-I was shown in 114 healthy children and adolescents to both correlate with height and reflect spontaneous GH secretion [4]. IGF-I levels are monitored during treatment with exogenous GH to assess adherence and inform dose adjustments [2,3,5]. Conversely, IGF-I levels are also employed in diagnosing and evaluating treatment efficacy for acromegaly, the state of excessive GH secretion [6,7].

Altered nutritional status represents the major non-GH, clinically relevant regulator of IGF-I levels [8]. Inadequate nutrition causes hepatic GH insensitivity, with a primary reduction in IGF-I production despite normal or even elevated levels of GH from loss of the normal IGF-I negative feedback on the pituitary gland and hypothalamus. This has been characterized in patients with anorexia nervosa [9,10], but also can be seen due to malnutrition from food insecurity (e.g., marasmus and kwashiorkor), milder dietary intake inadequacy that fails to meet daily demands, or specific micronutrient deficit like zinc deficiency [8]. Gastrointestinal diseases like Crohn’s disease, celiac disease, cystic fibrosis, and gastroesophageal reflux disease also can lower circulating IGF-I levels even in the absence of gastrointestinal symptoms [8]. In the other extreme, obesity blunts GH secretion, yet circulating IGF-I concentrations in obese individuals often are maintained or even higher than in non-obese controls. Although obesity did not associate with higher total IGF-I levels in multiple studies (indeed, an inverse U-shaped association between IGF-I z-score and body mass index (BMI) was shown in a population-based study of more than 6000 adults [11]), IGF-I bioavailability is increased [8].

Circulating IGF-I concentrations are dependent on age, gender, and gonadal status (pubertal status in adolescence, reproductive function in adults, and even whether estrogen replacement in women is administered orally versus transdermally) [12]. Normal IGF-I concentrations rise from infancy through childhood, peak during puberty (the pubertal growth spurt), and gradually decline throughout adulthood [13,14]. Alterations in circulating IGF-I concentration can also result from hepatic disease, renal dysfunction, and diabetes mellitus. Even controlling for these factors, various IGF-I assays often produce discordant results [15], leading to calls for harmonization of IGF-I assays to prevent diagnostic misclassifications and to allow meaningful inter-study comparisons of results in the literature [2,3,16]. 

Although closely related to IGF-I, IGF-II shows a different pattern. In healthy newborns, IGF-II concentrations are highest in the fetus, about half that of adult levels, which are reached by 1 year of age and persist through life [17]. In contrast, rodent IGF-II expression declines early postnatally, such that rodent models cannot serve to elucidate the physiologic function of IGF-II persistence in humans. Nonetheless, it is clear IGF-II plays an important role, especially in prenatal growth. In humans, the *IGF2* gene is imprinted and paternally expressed. DNA hypomethylation in the region of the *IGF2* gene that reduces paternal *IGF2* expression presents clinically with Silver–Russell syndrome, which is characterized by both prenatal and postnatal growth failure, often with body segment asymmetry [18]. *IGF2* overexpression (biallelic expression from relaxation or loss of imprinting) can result in Beckwith–Wiedemann syndrome, an overgrowth syndrome that also affects both pre- and postnatal growth, can include disproportionate growth (such as macroglossia and hemihypertrophy), and is associated with increased risk of embryonal tumors [19]. 

## 3. Molecular Regulation of IGF-I Production

IGF action can be modulated at the level of hormone (or autocrine/paracrine) production, hormone bioavailability, or receptor density and activity. Transcription of the *Igf1* gene, on chromosome *12q23.2*, is regulated by GH, in a mechanistic axis termed the “somatomedin hypothesis”, which has undergone considerable refinement over the years [20,21]. The GH receptor is a paradigmal cytokine receptor, whose activation recruits the tyrosine kinase Janus kinase 2 (JAK2). This, in turn, activates the signal transducers and activators of transcription, especially STAT5b [22], a transcription factor that stimulates transcription of the IGF genes. Apart from JAK2, the GH receptor also directly activates the Src tyrosine kinase pathway, the MAP kinase pathway, the PI3K/Akt pathway, and the mTOR pathway [23,24,25]. Naturally occurring and experimentally induced mutations have shed light on the specificity of second messenger recruitment and the specificity of outcomes conferred by them. Mutations in the GH receptor or in STAT5b are known to impair IGF production and lead to patients with primary IGF-deficient growth failure [26,27]. 

The *Igf1* gene encodes the 7.6 kD, single chain 70 amino acid polypeptide, that is cross-linked by disulfide bridges [28]. The *Igf2* gene, on chromosome 11p15.5, encodes the single chain 67 amino acid polypeptide [29] and is primarily regulated by imprinting. As discussed below, IGF-I and IGF-II production is determined not only by their transcriptional regulation, but also by interactions with dedicated molecular chaperones.

IGF bioavailability is primarily regulated via a family of six high-affinity IGF binding proteins (IGFBPs). Additional lower-affinity IGF binding proteins (named IGFBP-related proteins (IGFBPrPs)) were found by in silico searches for homology to the known IGFBPs; many of these molecules were previously known in other contexts, serving roles in normal or neoplastic growth [30]. The IGFBPs prolong the circulating half-life of IGF, transport the IGFs to target cells, and modulate the interaction of the IGFs with their surface membrane receptors via competitive inhibition. Local proteases, such as metalloproteinase pregnancy associated plasma protein A2 (PAPPA2), cleave the IGFBP, releasing the IGF for binding and activation of its receptor [31]. Of note, the IGFBPs have been found to perform various IGF-independent functions as well [30]. 

The actions of both IGF-I and IGF-II is mediated via the type 1 IGF receptor (IGF1R), an α2β2 transmembrane tyrosine kinase receptor that upon ligand binding, autophosphorylates and phosphorylates signaling pathways such as MAPK and PI3K/Akt [32]. IGF1R bears a high degree of homology to the insulin receptor, and αβ-hemireceptors of the two can form functioning hybrid receptors [33]. IGF1R signaling is regulated by internalization of bound receptors into clathrin-coated pits [34]. Phosphatases like SHP2 also can limit IGF1R signaling [35]. In contrast to IGF1R, the type 2 IGF receptor binds only IGF-II with high affinity, does not possess any recognizable signal transduction mechanism, and is identical to the cation-independent mannose-6-phosphate (CIM6P) receptor, a protein involved in intracellular lysosomal targeting [36]. Given the complexities of the system, an IGF-IR kinase receptor activation assay has been developed to measure IGF-1R stimulating activity (phosphorylation of tyrosine residues of the IGFIR) as a means of assessing the net effects of the system’s multiple players in various conditions [37]. 

Whereas the transcriptional and translational regulation of IGF production follow usual paradigms, the post-translational regulation of IGFs has unique features. First, as discussed below, maturation of IGF-I depends on the activity of GRP94, and without it IGF-I does not complete its biosynthesis and is not secreted [38]. This chaperone interaction provides a new element of regulation outside the “standard” GH system. Second, as mentioned above, the complexes of IGF-I with the binding proteins are important for IGF-I function.

## 4. GRP94

**Figure 1:**
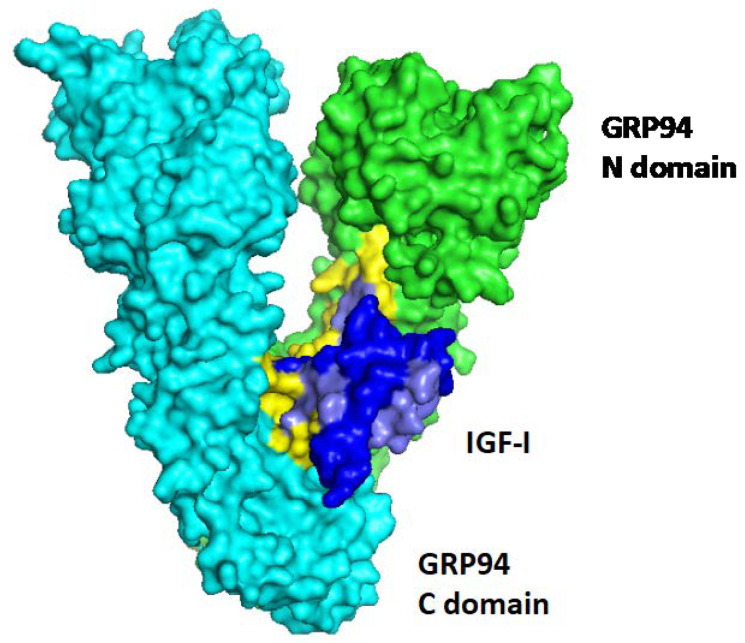
A predicted complex between GRP94 and IGF-I. The crystal structure of human IGF-1 (1IMX [63]) was docked onto the crystal structure of GRP94 (2O1U [64]) with the ZDOCK algorithm (version 3.0.2 [65]). The two monomers of GRP94, are shown in cyan and green, with the N-terminal and C-terminal domains indicated. The interacting amino acids are colored yellow. The complex shown is the highest scoring predicted complex, and eight other complexes out of the 10 highest scoring ones overlap with it, predicting the same topology of binding. The GRP94 interacting residues are from from the internal face of the Middle domain and the C-terminal domain of the chaperone. The IGF-I interacting residues are mostly derived from its N-terminal 32 amino acids, colored light blue, while the C-terminal 28 residues (deep blue) are mostly predicted as non-interacting.

GRP94 is a glucose-regulated protein of 94 kDa molecular size, encoded by the gene *HSP90B1* (*OMIM #*191175), whose chromosomal location is remarkably close to the IGF-I gene. Its expression is ubiquitous and its transcription is upregulated by low glucose tension [39], among other conditions. GRP94 also is commonly known as gp96, ERp99, or endoplasmin [40], referring to its extensive glycosylation and its abundance in the endoplasmic reticulum. It has the domain structure typical of the heat shock 90 (HSP90) family of proteins, including a C-terminal domain that mediates the constitutive dimerization of GRP94 (Figure 1). Like all family members, the N-terminal domain of GRP94 is a typical ATP-binding domain [41] that affects the dimerization of GRP94 and its action cycle [42]. The N-terminal domain also mediates binding of antigenic peptides [43] through which GRP94 activates T cells, the basis for the immunological function of GRP94 [43,44]. The protein chaperone function of GRP94 also requires the N-terminal ATPase domain [45], but the protein binding site is thought to reside in the C-terminal domain, around residues 652–678 [46].

GRP94 is an essential chaperone for multiple receptors and secreted proteins [47] (Table 1). Protein interaction data using GRP94-sufficient and -deficient cells show physical interactions with ~200 proteins and effects on expression levels of ~500 proteins [48], including some of the verified substrates listed in Table 1. Much of this interactome remains to be characterized. For some of the substrates (e.g., IGFs) there is genetic evidence that GRP94 is essential, whereas others can be expressed properly (albeit at lower abundance) even absent GRP94. For many of the susbstrates (e.g., thyroglobulin), data only show physical association without a physiological conseqence.

As can be gleaned from this non-exhaustive listing, GRP94 substrates (also called “clients”) are found in a variety of tissues and cell types. These substrates share no common structural motif that would predict their association with GRP94, nor do they share a common protein fold or a characteristic post-translational modification, aside from internal disulfide bonds (Table 1). The only obvious common denominator is that the substrates are secreted or membrane-bound proteins that are made in the endoplasmic reticulum. Importantly, even in cases of verified GRP94-substrate interactions, there can be exceptional isoforms or family members that do not interact, for example, TLR3 vs. most other TLRs [49]. 

No GRP94–susbstrate complex has been purified and analysed so far, so the exact mode of interaction currently can only be simulated, as shown in Figure 1 with human IGF-I [62] and GRP94 [63], using the ZDOCK algorithm [64]. Furthermore, as GRP94, like all chaperones, binds substrates that have not yet reached their final three-dimensional structure, the precise interaction is only approximated based on known mutations in the interacting proteins. This is an implicit limitation of docking studies such as that shown in Figure 1. 

Along with molecular specificity, another enigmatic feature of GRP94 is the paucity of co-chaperones. Its cytosolic homolog, HSP90, has a well-known set of auxiliary proteins that form transient complexes and impact the quality and/or speed of enhanced folding of the respective substrates [65]. Even other types of ER chaperones have well known co-chaperones, some general for all substrates and some substrate-dedicated [66]. In contrast, GRP94 is currently known to work with only one co-chaperone, CNPY3 [49] (see below). As far as the insulin/IGF substrates, GRP94 co-chaperones are presumably yet to be characterized, because genetic data show that ASNA-1 is an evolutionarily conserved ATPase that is important for insulin/IGF maturation in both worms and mammals [67] (see the next section). Further characterization of co-chaperones will no doubt explain many of the unresolved details about the action cycle of the GRP94 chaperone machine.

GRP94 differs from the cytosolic HSP90 orthologs in inherent, functionally-relevant structural properties [42] such as the nucleotide-dependent conformational changes of the N-terminal domain [63] as well as the interactions mediated by the charged linker domain. These differences lead to a different action cycle of this protein [68,69] and probably also to its ability to chaperone folding of client proteins without the many co-chaperones that are required for activity of the cytosolic HSP90 orthologs [70]. 

## 5. GRP94 as an Obligate Chaperone for IGF-I and IGF-II Production

The dependence of IGF-I maturation and secretion on GRP94 is a property also exhibited by IGF-II [38,71] and insulin [58] (Table 1), and even by the insulin-like proteins of the nematode *C. elegans*, some of which have only weak primary sequence similarity to IGF-I [72], showing that it is evolutionarily conserved. In contrast, within the TLR family of substrates, TLR3 is exceptional in its refractiveness to GRP94, showing the selectivity of substrate selection by GRP94 [49]. The chaperone dependence of the IGFs is based on physical association of pro-IGFs (or pro-insulin) with GRP94, an association that is transient and occurs early during biosynthesis [38,73]. The precise amino acids of the pro-insulins that interact with GRP94 have not been mapped, but some experiments plus molecular modelling indicate that the pro-insulins do not bind at the site of GRP94 that is responsible for binding of antigenic peptides [43], but rather bind at a more distal site encompassing the middle and C-terminal domains of GRP94 [58]. Apparently, their binding site overlaps residues 652–678 [58], the region that was identified for binding integrins and TLRs [46]. Nonetheless, despite such overlap, there is more complex specificity built into client selection, for example, the pair Met658/Met662 residues are essential for integrin folding but not TLRs [46].

Folding of client proteins often involves not just a chaperone protein, but also recruitment of additional proteins dedicated for the client, which serve as co-chaperones. In the case of GRP94, the ER luminal protein CNPY3 binds to GRP94 when it is engaged in biosynthesis of Toll-like receptors, but not other clients. CNPY3 and GRP94 interact with each other and with the TLR client in nucleotide-dependent manner [49]. Similar complexes have not been defined for the IGF/insulin proteins, but they likely exist; ASNA1, for example, is an ATPase expressed in insulin/IGF-producing cells in both worms and humans which regulates insulin secretion [67]. 

The biological importance of the IGF–GRP94 interaction is highlighted by the discovery of a hypomorphic variant of human GRP94, P300L, that affects the IGF chaperone activity and limits IGF biosynthesis [74]. Only four homozygotes have been identified so far, a lower frequency than expected from genetic principles [74], and heterozygous carriers of P300L are a noncommon single nucleotide polymorphism with frequencies of 1–4% in various populations. Carriers have 9% lower circulating IGF-1 concentration. In cell models of P300L heterozygosity, half as much IGF was secreted relative to wild type GRP94 [74]. It should be noted that the marked dependence of IGFs on GRP94 activity is unusual—depletion of the chaperone has much milder effects on the expression of some GRP94 client proteins compared to the secretion of the insulin family clients [52]. 

Why does the insulin/IGF structure require GRP94? At present, this question is not properly answered, and the available data only provide hints. The insulin-like family of proteins is unusual in that they are made initially as small (less than 100 amino acids) pro-proteins, that are processed proteolytically [72,75]. Furthermore, most of these sequences encode for three disulfides [72] that need to be bonded in a precise order within a small molecular space, a considerable folding challenge [76,77]. The surprising finding that at least one IGF-I variant has alternative folded states [78] underscores the folding difficulty, which is one likely reason for the need for molecular chaperones. As GRP94 has been found to interact with PDIs [60,79] it may act as a scaffolding protein in the recruitment of PDIs during the folding of the substrates [80].

The essential chaperoning role of GRP94 towards IGFs has implications for cell growth, for normal tissue differentiation and for cancer progression. A common cellular stress situation is the withdrawal of growth factors from cells, many of which respond to such stress by autocrine production of the growth factors [81]. However, cells with mutated or drug-inhibited GRP94 cannot produce these growth factors [38], leading to arrested growth/differentiation and, in extreme cases, cell death. The requirement for functional GRP94 in development is illustrated by the dramatic impact of tissue-specific GRP94 depletion on striated muscle [57], where myotube fusion and expression of contractile proteins downstream of the master MyoD transcription program are inhibited, coincident with the known need for synergistic input from growth factor signaling [73]. In cancer, elevated expression of GRP94 is observed in melanoma, ovarian cancer, multiple myeloma, lung cancer, and inflammation-associated colon cancer. GRP94 expression in cancer cells is closely linked to cancer growth and metastasis through a number of its clients, as listed above [82]. In part, this is due to response of the GRP94 promoter to some aspects of the tumor microenvironment that may include low glucose level [83], but is distinct from hypoxia [84]. The increase in GRP94 expression in tumors is tightly linked to their increased cellular proliferation rate and migration capacities and to their increased production of growth factors [85].

Constitutive overexpression of GRP94 is a common survival pathway that is usually used during oxidative stress [86], reflecting the many pathways that involve GRP94. The above three examples highlight situations that upregulate GRP94 more specifically, because of subsets of interacting proteins. 

Association with GRP94 is by no means the only protein–protein interaction that IGF-I undergoes. Circulating IGF-I is secreted mainly by the liver and circulates bound to IGF-binding proteins (IGFBPs), either as binary complexes or ternary complexes primarily with IGFBP-3 or IGFBP-5 and an acid-labile subunit (ALS). The components of these circulating complexes are produced by different cells and the complexes assemble after secretion to the circulation [87]. The complexes are important for the stability of circulating IGFs and also for their signaling function; in the absence of IGFBPs, there is much lower level of serum IGF-1, but surprisingly, this neither predicted growth potential or skeletal integrity nor defined GH secretion or metabolic abnormalities [88]. 

Each IGF-I associated protein appears to play a distinct role in determining musculoskeletal phenotype, with different effects on cortical and trabecular bone compartments and the striated muscles [88,89]. The differential effects of hepatic vs. autocrine/paracrine IGF-I is likewise attributable to different complexes, either due to differential assembly or to different proteases at the target tissue that cleave the IGFBP to release IGF-I to interact with IGF1R, IGF1R and insulin receptor density, etc. [90]. Similarly, when skeletal muscle deletion of GRP94 is used to limit production of IGF-I, endocrine and paracrine IGF-I are shown to regulate both tissue growth and body plan [57,88].

## 6. Conclusions

### 6.1. Implications for Novel Mechanisms of Idiopathic Short Stature

The novel association of IGFs with GRP94 that modulates production of IGFs has two implications for idiopathic short stature and other growth deficiencies. First, as allelic variations of the chaperone are likely to be new determinants of stature, there are now new target genes that can be screened to explain clinical observations. Second, based on other interacting proteins like ASNA1, we expect that the production of multiple insulin-related proteins will be sensitive to the activity of these proteins, in addition to the quality of the insulin-related protein itself. The chaperone machinery can be modulated with small molecules, so either GRP94 itself or its interacting proteins provide a novel way to manipulate both IGF deficiency and excessive production.

### 6.2. Implications for Cancer Treatment 

The IGF-GRP94 interaction has similar implications for cancer, suggesting a potential role for both genetic screening for and pharmacological agents against the GRP94 machinery. Tumors often conscript IGF system overactivity as a means of furthering the neoplastic process. Autocrine/paracrine IGF overexpression by tumor cells or supporting stromal cells serves to stimulate cancer progression. As an obligate chaperone for secretion of both IGF-I and IGF-II, GRP94 may become a novel target for anti-neoplastic therapy. This may be particularly important for cancers like breast and prostate that become IGF-dependent when they become sex hormone-independent. It is conceivable that differences in the association of IGF-I and IGF-II with GRP94 can be exploited for selective tissue targeting of compounds and it is also possible that distinct, tissue-specific auxiliary proteins are involved in complex formation in different cells and therefore can be targeted selectively. 

## Figures and Tables

**Table 1:** Protein substrates of GRP94.

Protein Substrate	Refs	Major Expression	Notable Structural Features
Immunoglobulin L chain H chain	[50,51]	B lineage cells	Immunoglobulin fold Non-glycosylated secreted Glycosylated secreted or membrane-spanning
Toll-like receptor	[52,53]	Ubiquitous, predominantly leukocytes	Leucine-rich repeats; Membrane-spanning proteins
Integrins	[48]	Ubiquitous	Immunoglobulin superfamily membrane-spanning heterodimers
LRP6	[54,55]		EGF-like repeats β-propeller motifs Interacts indirectly via MesD
Glycoprotein Ib-IX-V complex	[56]	Platelets	
Insulin-like proteins IGF-I IGF-II Insulin	[38,57,58]	Ubiquitous Pancreatic β cells	
Thyroglobulin	[59,60]	Thyrocytes	Large disulfide-bonded protease-type repeats
GARP	[61]	Treg cells; Platelets	Membrane-spanning leucine-rich repeats domains Tregs and platelets
